# Association of bilaterally suppressed EEG amplitudes and outcomes in critically ill children

**DOI:** 10.3389/fnins.2024.1411151

**Published:** 2024-06-05

**Authors:** Luisa Paul, Sandra Greve, Johanna Hegemann, Sonja Gienger, Verena Tamara Löffelhardt, Adela Della Marina, Ursula Felderhoff-Müser, Christian Dohna-Schwake, Nora Bruns

**Affiliations:** ^1^Department of Pediatrics I, Neonatology, Pediatric Intensive Care Medicine, Pediatric Neurology, and Pediatric Infectious Diseases, University Hospital Essen, University of Duisburg-Essen, Essen, Germany; ^2^C-TNBS, Centre for Translational Neuro-and Behavioural Sciences, University Hospital Essen, University of Duisburg-Essen, Essen, Germany; ^3^Department of Pediatric Cardiology/Congenital Cardiology, Heidelberg University Medical Center, Heidelberg, Germany

**Keywords:** amplitude-integrated EEG, pediatric intensive care unit, children, neurocritical illness, mortality, PCPC, outcome, amplitude suppression

## Abstract

**Background and objectives:**

Amplitude-integrated EEG (aEEG) is used to assess electrocortical activity in pediatric intensive care if (continuous) full channel EEG is unavailable but evidence regarding the meaning of suppressed aEEG amplitudes in children remains limited. This retrospective cohort study investigated the association of suppressed aEEG amplitudes in critically ill children with death or decline of neurological functioning at hospital discharge.

**Methods:**

Two hundred and thirty-five EEGs derived from individual patients <18 years in the pediatric intensive care unit at the University Hospital Essen (Germany) between 04/2014 and 07/2021, were converted into aEEGs and amplitudes analyzed with respect to age-specific percentiles. Crude and adjusted odds ratios (OR) for death, and functional decline at hospital discharge in patients with bilateral suppression of the upper or lower amplitude below the 10th percentile were calculated. Sensitivity, specificity, positive (PPV) and negative predictive values (NPV) were assessed.

**Results:**

The median time from neurological insult to EEG recording was 2 days. PICU admission occurred due to acute neurological diseases in 36% and patients had high overall disease severity. Thirty-three (14%) patients died and 68 (29%) had a functional decline. Amplitude suppression was observed in 48% (upper amplitude) and 57% (lower amplitude), with unilateral suppression less frequent than bilateral suppression. Multivariable regression analyses yielded crude ORs between 4.61 and 14.29 and adjusted ORs between 2.55 and 8.87 for death and functional decline if upper or lower amplitudes were bilaterally suppressed. NPVs for bilaterally non-suppressed amplitudes were above 95% for death and above 83% for pediatric cerebral performance category Scale (PCPC) decline, whereas PPVs ranged between 22 and 32% for death and 49–52% for PCPC decline.

**Discussion:**

This study found a high prevalence of suppressed aEEG amplitudes in critically ill children. Bilaterally normal amplitudes predicted good outcomes, whereas bilateral suppression was associated with increased odds for death and functional decline. aEEG assessment may serve as an element for risk stratification of PICU patients if conventional EEG is unavailable with excellent negative predictive abilities but requires additional information to identify patients at risk for poor outcomes.

## 1 Introduction

Acute neurological illness accounts for up to 16% of pediatric intensive care unit (PICU) admissions (Fink et al., 2017; Williams et al., 2018) and an unknown proportion of PICU patients is at risk of secondary neurologic deterioration with subsequent neurologic sequelae. Evidence-based serial or continuous assessment of neurological function in critically ill children is indispensable to direct neuroprotective interventions, to avoid and reduce neurologic sequelae, and preserve the developmental potential of each patient (Brown et al., 2022).

The gold standard to assess electrocortical activity is continuous full-channel EEG but suffers from high barriers to implementation even in high-resource settings (Ghossein et al., 2021). To facilitate the detection of changes of EEG activity despite the absence of EEG-trained personnel, mathematical transformations of raw EEG curves are increasingly applied in intensive care settings (Haider et al., 2016; Lalgudi Ganesan et al., 2018; Hwang et al., 2022). These transformations provide time-compressed quantitative trend information on specific features, such as amplitude, frequency, symmetry or burst rate and are summarized with the term quantitative EEG (qEEG). The oldest type of qEEG is the amplitude-integrated EEG (aEEG), which display the height of amplitudes (Maynard et al., 1969). After filtering and processing of the raw EEG signal, the highest and lowest amplitudes within a given time period are graphed on a semilogarithmic scale, creating a characteristic band. Via time-compressed depiction, several hours of electrocortical activity are depicted in the same display, enabling identification of subtle amplitude changes over time (Maynard et al., 1969; Bruns et al., 2017). aEEG can be used with any set of electrodes but in everyday clinical practice is commonly applied with a reduced set of electrodes especially in neonates and preterm infants.

Suppressed aEEG amplitudes are associated with poor outcomes in preterm infants, asphyxiated full-term neonates, and adults after cardiac arrest (Bruns et al., 2016; Sugiyama et al., 2016, 2018; Goeral et al., 2017). In children, however, the general level of evidence regarding the utility of aEEG is still low (Bruns et al., 2021). While reference values for healthy children were published recently (Greve et al., 2022; MacDarby et al., 2022), there is only incipient evidence from small studies that amplitude suppression may be associated with death or unfavorable outcomes as shown in neonates and adults (Bruns et al., 2019; Bourgoin et al., 2020; Beck et al., 2022). Yet, aEEG devices are broadly available in pediatric departments in Europe due to their indispensable role for the diagnostic and therapeutic decision-making path in neonatal asphyxia (Bruns et al., 2020). Pediatric intensive care teams in Europe become increasingly familiar with aEEG devices, promoting this type of EEG monitoring until full channel EEG or as an adjunct to (continuous) full channel EEG (Bruns et al., 2020; Butler et al., 2021).

The aim of this study was to assess the association of suppressed aEEG amplitudes in critically ill children with death and functional neurological short-term outcome. We retrospectively processed aEEGs from conventional EEGs that were conducted in our PICU and investigated the height of amplitudes as a predictor for death or functional decline at hospital discharge.

## 2 Methods

### 2.1 Study setting and eligibility

This is a retrospective observational cohort study on children admitted to the PICU of the University Hospital Essen (Germany) who received a full channel EEG recording during their PICU stay. To identify eligible patients, the EEG database of the children’s hospital was screened for EEGs conducted in the PICU since the database implementation in April 2014 until July 2021.

All EEGs conducted in patients <18 years in the PICU during the study period were assessed for eligibility. If a patient received several EEGs during the same PICU stay, only the first EEG was eligible for analysis.

### 2.2 Clinical data and outcomes

Clinical data were retrieved by review of digitalized patient charts and from the electronic patient documentation system.

– Acute disease severity at PICU admission was quantified by using the pediatric sequential organ failure assessment (pSOFA) (Matics and Sanchez-Pinto, 2017). Patients with pSOFA scores above >8 are considered at high risk for mortality.
– Acute neurological injuries were identified from cranial imaging findings: Focal lesions were graded based on the classification by Firsching et al. (2001) with slight modifications. Generalized edema was graded by severity as indicated in the written radiologist report (Supplementary Table S1). Acute neurological disease was categorized by etiology and type into generalized and focal disease (Supplementary Table S1).
– Sedation levels were categorized based on the substance, dosage, and type of administration (continuous versus intermittent) within the last 24 h before the EEG recording (Supplementary Table S2).
– Functional neurological status before acute critical illness and at hospital discharge was determined by the pediatric cerebral performance category (PCPC) (Fiser, 1992). Neurological outcome was categorized as good (PCPC decline from baseline to discharge <2) or poor (PCPC decline ≥2 or death).
– Death was defined as in-hospital death during the same hospital stay.

### 2.3 Local practice of EEG recording

In our PICU, full channel EEG is available only at daytime on regular working days. The target duration of EEGs is at least 20 min. During off-hours, continuous reduced channel EEG and aEEG is conducted in acute neurologically ill patients or those at risk for neurological complications. In patients with continued neurological deficits, coma, or high risk for unfavorable neurological outcomes, the reduced channel EEG is interrupted to enable full channel conventional recording EEG after reduction/cessation of sedative agents. All EEGs analyzed for this study were recorded as full channel conventional EEGs.

### 2.4 EEG conduction and aEEG conversion

Full-channel EEGs were conducted according to the international 10–20 system after skin preparation with OneStep EEG Gel Abrasiv plus^®^. Impedance was checked and skin preparation repeated until impedances were < 5 kΩ for all electrodes, as required by the German Society for Clinical Neurophysiology and Functional Imaging. All EEGs were recorded using Neurofax EEG devices and polaris.one software v4.0.4.0 (Nihon Kohden, Tokyo, Japan). aEEGs were computed from entire recordings with Polaris Trend Software QP-160AK (Nihon Kohden, Tokyo, Japan). Sections with artifacts were marked via artifact detection by the software.

### 2.5 Amplitude assessment

The C3-P3 and C4-P4 channels of the converted aEEG were analyzed across the entire recording semi-automatically by two independent researchers (LP and NB) as previously described (Greve et al., 2022; Löffelhardt et al., 2022). Briefly, amplitudes were measured by aligning the integrated measurement tool with the upper and lower amplitude. Next, the mean value between the two raters was calculated and used for further calculations and analyses. Sections with artifacts (as indicated by the EEG software) were ignored.

Based on previously specified age-specific percentiles for awake children (Greve et al., 2022), the upper and lower amplitudes were classified as suppressed (<10th percentile) or normal (≥10th percentile) for each hemisphere. Suppression patterns were then classified into normal (no amplitude suppressed), unilateral amplitude suppression, or bilateral amplitude suppression for the upper and lower amplitudes. Sleep/wakefulness during the recording was not accounted for in the regression model.

### 2.6 Missing data

There were no missing data on patient demographics, mortality, and PCPC. Missing data in predictor variables (pSOFA, radiological findings) was rare. These cases were handled as follows: Missing components of the pSOFA were assumed to be normal as suggested in the original publication of the score (Matics and Sanchez-Pinto, 2017). If no cranial imaging was available, findings were assumed as normal. There was no missing information on neurological diagnoses. All analyzed cases had complete data sets for predictor and outcome variables.

### 2.7 Adjustment sets for multivariable regression analyses

Minimally sufficient adjustment sets (MSAS) for multivariable regression analyses were identified using causal diagrams based on the theory of directed acyclic graphs (DAG) (Pearl, 1995; Greenland et al., 1999). DAGs are recommended for derivation of MSAS for empirical research in pediatrics and intensive care medicine (Williams et al., 2018; Lederer et al., 2019). The MSAS is derived based on the causal relationship and interaction between potential covariates. In this study, the MSAS comprised the type of acute neurologic injury, sedation levels at the time of recording, acute overall disease severity (pSOFA score), and neurological functional status before the acute critical illness (PCPC) (Supplementary Figure S1). The type of acute neurologic injury included the location and the etiology (Supplementary Table S1).

### 2.8 Statistical analyses

Descriptive analyses are presented as mean ± standard deviation (SD) for continuous normally distributed data; skewed data are presented as median and interquartile range (IQR). Discrete variables are summarized as counts and relative frequencies.

Interrater agreement was assessed by Bland-Altmann plots (Bland and Altman, 1986) and intraclass correlation coefficients [two-way mixed model for individual ratings (ICC 3,1)] (Shrout and Fleiss, 1979) for the upper and lower amplitudes as previously described (Greve et al., 2022; Löffelhardt et al., 2022).

Adjusted and unadjusted odds ratios with 95% confidence intervals (CI) for death and functional decline were calculated by multivariable logistic regression analyses comparing patients with no versus bilateral amplitude suppression. Areas under the curve (AUC) were calculated to assess the discrimination of the models and Hosmer-Lemeshow tests were conducted to assess the calibration. Patients with unilateral amplitude suppression were excluded from regression analyses. Likewise, sensitivity, specificity, and positive and negative predictive values were calculated for no versus bilateral amplitude suppression.

SAS Enterprise Guide 8.3 (SAS Institute Inc., Cary, NC, United States) was used for statistical analyses and figure production.

### 2.9 Ethics approval

The study was approved by the local Ethics Committee of the Medical Faculty of the University of Duisburg-Essen (21-10264-BO). Written informed consent was waived according to local legislation for analysis of retrospective anonymized data.

## 3 Results

**Figure 1:**
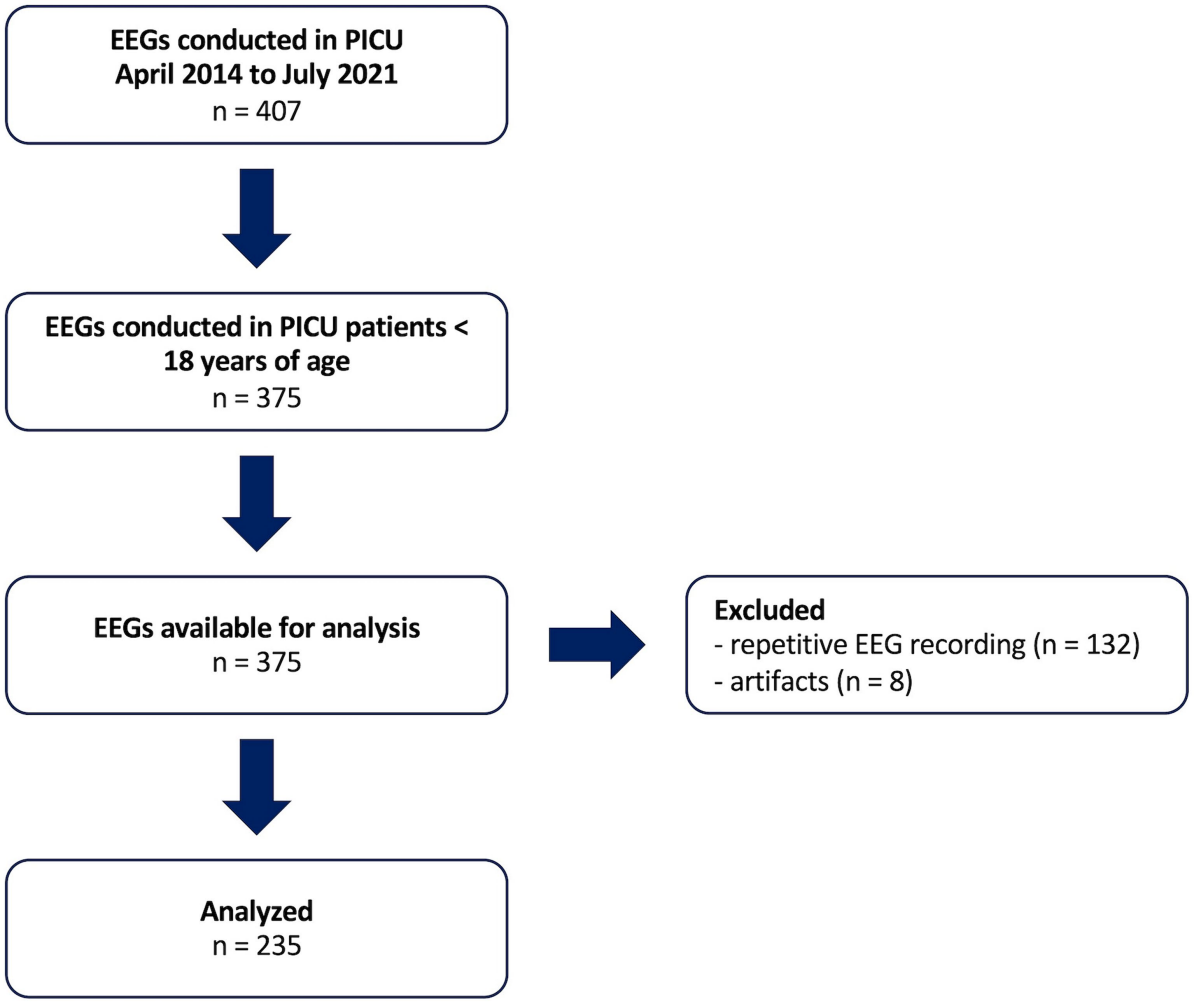
Screened and analyzed EEGs. PICU, pediatric intensive care unit.

In total, 235 EEGs from 235 individual patients with a mean duration of 16:46 min (±6:15 min) were analyzed (Figure 1), which is shorter than the target duration at our department. The median time between neurological insult and PICU admission was 0 days and median time from neurological insult to EEG recording was 2 days. The included patients displayed high degrees of acute overall and neurological illness with median pSOFA scores of 4 (interquartile range 2–7) and neurological reasons for PICU admission in 117 cases (50%) (Table 1). Thirty-three (14%) patients died and 68 (29%) displayed functional declines. Patients with good outcome were older, had lower pSOFA scores, received mechanical ventilation and sedation less frequently, and had shorter durations of PICU and hospital stay. Across groups, most children had previously good neurological function with baseline PCPC scores differing only in the upper quartiles.

**Table 1:** Patient characteristics by outcome group.

Patient characteristics	Outcome		
	Good (*n* = 167)	Poor (*n* = 68)	Deceased* (*n* = 33)
Female	69 (41%)	27 (39%)	13 (39%)
Age (years), median (IQR)	3 (0–11)	2 (0–7.5)	0 (0–5)
Weight (kg), median (IQR)	15.0 (7–40)	11.5 (6.4–24)	10.0 (6.0–19.4)
**Amplitude suppression**			
Upper amplitude, unilateral	26 (16%)	12 (18%)	4 (12%)
Upper amplitude, bilateral	38 (23%)	36 (53%)	24 (73%)
Lower amplitude, unilateral	38 (23%)	4 (6%)	2 (6%)
Lower amplitude, bilateral	46 (28%)	46 (68%)	28 (85%)
**Reason for PICU admission**			
Acute neurologic disease	62 (37%)	23 (33%)	6 (18%)
Cerebral injury or lesion (trauma, bleeding, tumor)	11 (7%)	13 (19%)	6 (18%)
Hypoxia/resuscitation	2 (1%)	6 (9%)	5 (15%)
Organ failure (non-neurologic)	30 (18%)	9 (13%)	7 (21%)
Other	62 (37%)	17 (25%)	9 (27%)
Days between PICU admission and insult, median (IQR)	0 (0–0)	0 (0–0)	0 (0–0)
Days insult to EEG, median (IQR)	2 (1–5)	3 (1–6)	2 (1–6)
pSOFA score	4 (2–6)	6.5 (2–10)	9 (6–11)
Cardiopulmonary resuscitation prior to EEG	10 (6%)	15 (22%)	11 (33%)
Invasive mechanical ventilation, *n* (%)	32 (19%)	42 (61%)	28 (85%)
Administration of sedative agents within 24 h before or during recording	57 (34%)	49 (72%)	24 (73%)
Sedation by continuous infusion	45 (27%)	44 (65%)	24 (73%)
Antiepileptic drug treatment at time of EEG**	27 (16%)	12 (18%)	2 (6%)
PCPC baseline, median (IQR)	1 (1–2)	1 (1–1.5)	1 (1–2)
PCPC ICU discharge, median (IQR)	1 (0–1)	5 (4–6)	6 (6–6)
PCPC hospital discharge, median (IQR)	2 (1–2)	5 (3–6)	6 (6–6)
PCPC decline baseline to hospital discharge, median (IQR)	0 (0–1)	3 (2–5)	5 (4–5)
Length of PICU stay (days), median (IQR)	10 (4–21)	16 (5–26)	9 (3–19)
Length of hospital stay (days), median (IQR)	17 (9–35)	26 (10–42)	9 (3–20)
Death, *n* (%)	0 (0%)	33 (49%)	33 (100%)
Death from neurological disease, *n* (%)	0 (0%)	24 (35%)	24 (73%)

If not otherwise indicated, numbers are presented as *n* (%).

*Subset of poor outcome group; **In addition to sedation; IQR, interquartile range; PICU, pediatric intensive care unit; pSOFA, pediatric sequential organ failure assessment.

The intraclass correlation coefficients were 0.94 both for the upper and the lower amplitudes. The mean interrater difference for the upper amplitude was 0.2 μV (±8.0 μV) and 0.6 μV (±1.7 μV) for the lower amplitude.

Grouping by amplitude suppression pattern showed that amplitude depression below the 10th percentile was frequent (upper amplitude: *n* = 112, 48%, lower amplitude: *n* = 134, 52%) and that unilateral suppression was less frequent than bilateral suppression (Table 1).

Multivariable regression analyses were conducted for all patients with either bilaterally normal or bilaterally suppressed amplitudes, which were *n* = 197 for the upper amplitude and *n* = 193 for the lower amplitude. Crude ORs ranged between 4.61 and 14.29 for death and functional decline if upper or lower amplitudes were bilaterally suppressed (Table 2). Adjusted ORs were lower and ranged between 2.55 and 8.87. The discrimination assessment of the adjusted models showed AUCs above 0.87 and Hosmer-Lemeshow tests were non-significant for all adjusted models (Table 2).

**Table 2:** Adjusted and unadjusted odds ratios to predict outcomes and discriminatory model performance.

Outcome		Adjusted			Unadjusted		Amplitude	Odds ratio (95% CI)	AUC	HL test (p)	Odds ratio (95% CI)	AUC
In-hospital death	Upper	7.37 (2.21–24.60)	0.91	0.93	11.33 (4.09–31.37)	0.77
	Lower	8.87 (2.26–34.80)	0.88	0.83	14.29 (4.17–48.95)	0.75
PCPC decline ≥ 2	Upper	4.09 (1.71–9.81)	0.87	0.18	4.88 (2.52–9.45)	0.69
	Lower	2.55 (1.08–6.02)	0.87	0.07	4.61 (2.40–8.86)	0.68

AUC, area under the curve; CI, confidence interval; HL test, Hosmer-Lemeshow test.

The sensitivity to predict death via bilateral amplitude suppression was 0.83 for the upper and 0.90 for the lower amplitudes. To predict PCPC decline the sensitivity was 0.64 for the upper and 0.77 for the lower amplitudes. The specificity of bilaterally suppressed amplitudes to predict any of the target outcomes ranged between 0.60 and 0.73. Positive predictive values ranged between 0.30 and 0.52, whereas the negative predictive value was equal to or above 0.96 for death and above 0.83 for PCPC decline (Table 3).

**Table 3:** Sensitivity, specificity, positive and negative predictive values of bilateral amplitude suppression to predict outcome.

	In-hospital death		PCPC decline ≥ 2		Amplitude		Amplitude		Upper	Lower	Upper	Lower
Sensitivity (95% CI)	0.83 (0.69–0.97)	0.90 (0.80–1.00)	0.64 (0.52–0.77)	0.77 (0.67–0.88)
Specificity (95% CI)	0.70 (0.63–0.77)	0.60 (0.53–0.68)	0.73 (0.66–0.80)	0.66 (0.58–0.75)
PPV (95% CI)	0.32 (0.22–0.43)	0.30 (0.21–0.40)	0.49 (0.37–0.60)	0.52 (0.42–0.62)
NPV (95% CI)	0.96 (0.92–0.99)	0.97 (0.94–1.00)	0.84 (0.77–0.90)	0.86 (0.79–0.93)

CI, confidence interval; NPV, negative predictive value; PPV, positive predictive value.

## 4 Discussion

This study found suppression of EEG amplitudes in a high proportion of critically ill children. Suppressed amplitudes were associated with death and functional decline at discharge with high negative predictive values and low to moderate positive predictive values. Discrimination of outcomes in multivariable regression analyses was enhanced when baseline functional status, neurological lesions, acute disease severity, and sedation levels were accounted for. Overall, odds for death and functional decline were strongly increased if amplitudes were bilaterally below the 10^th^ percentile for age. Upper and lower amplitudes had roughly equal utility to predict or rule out target outcomes.

In children, abnormal EEG background and epileptogenic EEG activity after cardiac arrest have been linked to poor neurological outcomes (Anetakis et al., 2022; Smith et al., 2022; Bach et al., 2024). Discontinuous or burst suppression patterns are associated with worsened PCPC scores, mortality in children with electrographic status epilepticus (Topjian et al., 2013). Fung et al. found several variables, such as burst-suppression or attenuated-featureless EEG background to be associated with unfavorable neurobehavioral outcome in acute pediatric encephalopathy (Fung et al., 2021). However, it must be pointed out that these studies used continuous full channel EEG, a technique that requires expert review and is not commonly available even in non-low resource settings. Additionally, qEEG is used as an adjunct to full channel continuous EEG in some settings (Haider et al., 2016; Ng, 2017; Jiang et al., 2018; Lee et al., 2019). For that reason, aEEG serves as a bridging technology until EEG or as an adjunct to EEG and thus deserves further investigation regarding its potential to prognosticate outcomes.

This study confirms emerging evidence from three smaller studies that found an association between aEEG amplitudes and outcomes in PICU patients with meningitis and after cardiac arrest (Bruns et al., 2019; Bourgoin et al., 2020; Beck et al., 2022). The patient population in this study was highly preselected, with high overall disease severity and additionally high burden of neurologic disease compared to average PICU patients (Fink et al., 2017; Williams et al., 2018), putting these patients at special risk for death and neurologic sequelae. Our results suggest that aEEG may be helpful in identifying PICU patients at low risk for poor outcomes. Timely identification of non-low risk patients may warrant advanced neuromonitoring and potentially neuroprotective strategies and early neurorehabilitation in these patients. The differing discriminatory performance between adjusted and unadjusted regression models points out the importance of additional clinical information besides aEEG amplitudes. Of special importance in PICU patients, are sedation levels and neuroactive medication, which affect EEG activity (McKeever et al., 2012, 2014; Drohan et al., 2018; Schultz et al., 2022) and can induce severe amplitude suppression (Bruns, 2022; Falsaperla et al., 2022). Hemodynamic changes also affect electrocortical activity (Bruns et al., 2021), possibly via changes of oxygen supply and metabolic clearance.

A limitation of this study is that we assessed only the first EEG after PICU admission. Selection bias occurred from patients receiving high levels of sedation who therefore did not undergo EEG recording, or who died before EEG recording. The actual duration of EEG was shorter than the target duration of the department and the reasons for this are not clear. In this study, the short duration made it impossible to assess changes over time and may also have yielded less reliable measurements than a longer recording would have. On the other hand, this study shows that accompanying conditions are extremely important to account for. In the acute phase of critical illness, the interplay of vasopressor/inotrope administration, sedation levels, and organ dysfunction is highly dynamic, warranting advanced time series approaches to accurately account for changes of accompanying physiological and treatment conditions in long-term recordings. The “static” approach of this study made a large-scale aEEG assessment in very severely ill children possible for the first time, thereby providing important evidence for clinicians’ daily practice.

Another limitation of this study is the fact that by using the 10th percentile for awake children as a threshold to define amplitude suppression, 10% of healthy children physiologically display amplitudes that were classified as suppressed in this study. Compared to a lower percentile (e.g., 5th or 1st percentiles) as a threshold or the 10 μV cut-off suggested by the American Clinical Neurophysiology Society (ACNS) for post cardiac arrest EEGs in adults (Hirsch et al., 2013), our approach reduces specificity in favor of sensitivity. Because of the age-dependency of amplitude height (Greve et al., 2022; MacDarby et al., 2022) we decided against an absolute threshold like that of the ACNS. Sleep/wakefulness was not accounted for in this study. However, as amplitudes are higher during sleep than during wakefulness (Löffelhardt et al., 2022; MacDarby et al., 2022), sleep could not introduce bias from misclassification as suppressed aEEG. Larger scale studies should identify optimum thresholds to define amplitude suppression, ideally age-specific cut-off values. To optimize prediction and discrimination of death and functional outcome by EEG, further quantitative EEG parameters or a combination of several parameters should also be systematically investigated in this context. Due to the retrospective character of this study, the functional outcome assessment was limited to the PCPC as a simple and common measure of PICU outcomes. Prospective investigations should also assess cognitive, emotional, and social health functioning, as their impairment contributes to post-PICU sequelae, such as the post-intensive care syndrome (Manning et al., 2018; Perry-Eaddy et al., 2023; Rahmaty et al., 2023).

The most important finding of this study is that normal aEEG amplitudes were associated with good outcomes in critically ill children. Contrarily, bilateral amplitude suppression predicted mortality and functional decline with a specificity of approximately 70%. According to the results of regression analyses, aEEG interpretations need to account for neurological lesions, acute disease severity and neuroactive medication. Further research is needed to investigate even earlier aEEG recordings and the evolution of amplitudes over the course of disease. Further qEEG parameters should be examined regarding their potential to improve identification of high-risk patients and direct neuroprotective therapeutic and neuro-rehabilitative strategies.

## Data availability statement

The raw data supporting the conclusions of this article will be made available by the authors, without undue reservation.

## Ethics statement

The studies involving humans were approved by Ethics committee of the Medical Faculty of the University of Duisburg-Essen. The studies were conducted in accordance with the local legislation and institutional requirements. The ethics committee/institutional review board waived the requirement of written informed consent for participation from the participants or the participants’ legal guardians/next of kin because for retrospective anonymous data analysis no informed consent is required according to local law.

## Author contributions

LP: Investigation, Project administration, Supervision, Writing – original draft, Data curation. SGr: Data curation, Investigation, Writing – review & editing. JH: Investigation, Writing – review & editing, Data curation. SGi: Data curation, Investigation, Writing – review & editing. VL: Data curation, Investigation, Writing – review & editing. AD: Writing – review & editing, Conceptualization, Methodology, Supervision. UF-M: Writing – review & editing, Resources, Validation. CD-S: Resources, Validation, Writing – review & editing, Conceptualization. NB: Conceptualization, Formal analysis, Investigation, Methodology, Project administration, Software, Supervision, Visualization, Writing – original draft.
