# Correction: Association of bilaterally suppressed EEG amplitudes and outcomes in critically ill children

**DOI:** 10.3389/fnins.2025.1741863

**Published:** 2025-11-28

**Authors:** Luisa Paul, Sandra Greve, Johanna Hegemann, Sonja Gienger, Verena Tamara Löffelhardt, Adela Della Marina, Ursula Felderhoff-Müser, Christian Dohna-Schwake, Nora Bruns

**Affiliations:** 1 Department of Pediatrics I, Neonatology, Pediatric Intensive Care Medicine, Pediatric Neurology, and Pediatric Infectious Diseases, University Hospital Essen, University of Duisburg-Essen, Essen, Germany; 2 C-TNBS, Centre for Translational Neuro-and Behavioural Sciences, University Hospital Essen, University of Duisburg-Essen, Essen, Germany; 3 Department of Pediatric Cardiology/Congenital Cardiology, Heidelberg University Medical Center, Heidelberg, Germany

**Keywords:** amplitude-integrated EEG, pediatric intensive care unit, children, neurocritical illness, mortality, PCPC, outcome, amplitude suppression

There was a mistake in Table 1 as published. The corrected Table 1 appears below.

**Table 1:** Patient characteristics by outcome group.

	**Outcome**		
**Patient characteristics**	**Good (*****n*** = **167)**	**Poor (*****n*** = **68)**	Deceased^*^(***n*** = **33)**
Female	69 (41%)	27 (39%)	13 (39%)
Age (years), *median (IQR)*	3 (0–11)	2 (0–7.5)	0 (0–5)
Weight (kg), *median (IQR)*	15.0 (7–40)	11.5 (6.4–24)	10.0 (6.0–19.4)
**Amplitude suppression**			
Upper amplitude, unilateral	26 (16%)	12 (18%)	4 (12%)
Upper amplitude, bilateral	38 (23%)	36 (53%)	24 (73%)
Lower amplitude, unilateral	38 (23%)	4 (6%)	2 (6%)
Lower amplitude, bilateral	46 (28%)	46 (68%)	28 (85%)
**Reason for PICU admission**			
Acute neurologic disease	62 (37%)	23 (33%)	6 (18%)
Cerebral injury or lesion (trauma, bleeding, tumor)	11 (7%)	13 (19%)	6 (18%)
Hypoxia/resuscitation	2 (1%)	6 (9%)	5 (15%)
Organ failure (non-neurologic)	30 (18%)	9 (13%)	7 (21%)
Other	62 (37%)	17 (25%)	9 (27%)
Days between PICU admission and insult, *median (IQR)*	0 (0–0)	0 (0–0)	0 (0–0)
Days insult to EEG, *median (IQR)*	2 (1–5)	3 (1–6)	2 (1–6)
pSOFA score	4 (2–6)	6.5 (2–10)	9 (6–11)
Cardiopulmonary resuscitation prior to EEG	10 (6%)	15 (22%)	11 (33%)
Invasive mechanical ventilation, *n (%)*	32 (19%)	42 (61%)	28 (85%)
Administration of sedatives within 24 h before or during recording	57 (34%)	49 (72%)	24 (73%)
Sedation by continuous infusion	45 (27%)	44 (65%)	24 (73%)
Antiepileptic drug treatment at time of EEG^**^	27 (16%)	12 (18%)	3 (9%)
PCPC baseline, *median (IQR)*	1 (1–2)	1 (1–1.5)	1 (1–2)
PCPC ICU discharge, *median (IQR)*	1 (0–1)	5 (4–6)	6 (6–6)
PCPC hospital discharge, *median (IQR)*	2 (1–2)	5 (3–6)	6 (6–6)
PCPC decline baseline to hospital discharge, *median (IQR)*	0 (0–1)	3 (2–5)	5 (4–5)
Length of PICU stay (days), *median (IQR)*	10 (4–21)	16 (5–26)	9 (3–19)
Length of hospital stay (days), *median (IQR)*	17 (9–35)	26 (10–42)	9 (3–20)
Death, *n (%)*	0 (0%)	33 (49%)	33 (100%)
Death from neurological disease, *n (%)*	0 (0%)	24 (35%)	24 (73%)

If not otherwise indicated, numbers are presented as n (%). ^*^Subset of poor outcome group; ^**^In addition to sedation; IQR, interquartile range; PICU, pediatric intensive care unit; pSOFA, pediatric sequential organ failure assessment.

In the abstract, there was a misspelling in the sentence: “PICU admission occurred due to neurological reasons in 43% and patients had high overall disease severity.”

This has been corrected to read: “PICU admission occurred due to acute neurological diseases in 36% and patients had high overall disease severity.”

There was a misspelling in the sentence: “The included patients displayed high degrees of acute overall and neurological illness with median pSOFA scores of 4 (interquartile range 2–7) and neurological reasons for PICU admission in 156 cases (66%) (Table 1).”

A correction has been made to the section Results, first paragraph, and the corrected sentence reads: “The included patients displayed high degrees of acute overall and neurological illness with median pSOFA scores of 4 (interquartile range 2–7) and neurological reasons for PICU admission in 117 cases (50%) (Table 1)”.

The original version of this article has been updated.

