# Supplementary material for: Association of bilaterally suppressed EEG amplitudes and outcomes in critically ill children
Source: Front Neurosci. 2024 Jun 5;18:1411151. doi: 10.3389/fnins.2024.1411151 (PMC11188580; doi:10.3389/fnins.2024.1411151)
Supplement: Supplementary file 1 [file Data_Sheet_1.pdf]

Supplementary tables

Table S1: Grading of radiological findings and categorization neurological disease**

Radiological lesion grading**	Generalized		Focal	
	**Grade**		**Grade**	
	0	None	0	None
	1	Mild edema	1	Unilateral supratentorial
	2	Moderate edema	2	Bilateral or midline supratentorial
	3	Severe edema	3	Unilateral infratentorial
			4	Bilateral or midline infratentorial
**Clinical diagnosis category**	**Generalized**		**Focal**	
	None		None	
	Meningitis		Tumor	
	Encephalopathy		Hemorrhage	
	Seizure		Abscess/infection	
	Others		Cerebelitis	
			Others	

Adopted from Firsching et al.
*according to written radiologist report
**encountered imaging findings (in alphabetical order): aspergillosis, brain atrophy, cavernoma, cerebral calcification, cerebral edema, cerebral infarction, cerebral malformation, cerebral vascular anomalies, cerebral vascular dissection, empyema, encephalitis, encephalopathy, hemorrhage, hydrocephalus, hygroma, hypophysitis, hypoxic lesions, ischaemia, leukoencephalopathy, meningitis, necrosis, necrotising encephalitis, sinus vein thrombosis, skull fracture, subdural hematoma, tumor, unspecified hyperintensities, vasculitis

Table S2: Sedation grading

Grade	Administered sedation within 24 hours before /at time of EEG recording
0	No sedatives
1	SD only (benzodiazepine, propofol, ketamine, chloral hydrate) or procedure-associated continuous infusion of propofol within 24 hours before recording
2	Continuous infusion of opiate
3	Continuous infusion of opiate + benzodiazepine ± SD
4	Continuous infusion of opiate + benzodiazepine + ketamine ± SD
5	Inhalative sevoflurane / continuous infusion of thiopental

SD = single doses
