# Supplementary material for: Association of bilaterally suppressed EEG amplitudes and outcomes in critically ill children
Source: Front Neurosci. 2024 Jun 5;18:1411151. doi: 10.3389/fnins.2024.1411151 (PMC11188580; doi:10.3389/fnins.2024.1411151)
Supplement: Supplementary file 2 [file Image_1.pdf]

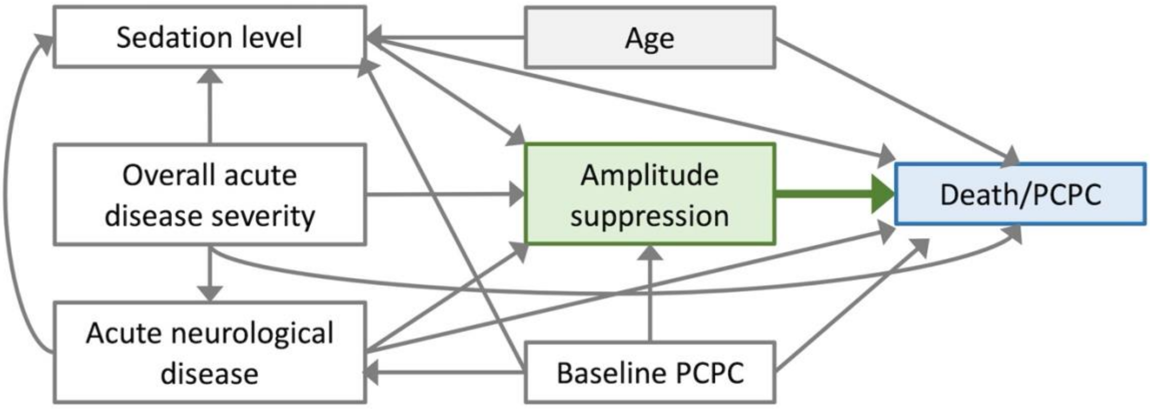

Figure S1: Causal diagram to identify the adjustment set for regression analyses. PCPC = Pediatric cerebral performance category. Box colors: green = exposure, blue = outcome, white = variables that must be adjusted for, grey = no adjustment necessary.
